# Thyroid autoimmunity and obstetric outcomes in women with recurrent miscarriage: a case–control study

**DOI:** 10.1530/EC-13-0012

**Published:** 2013-06-22

**Authors:** Kusum Lata, Pinaki Dutta, Subbiah Sridhar, Minakshi Rohilla, Anand Srinivasan, G R V Prashad, Viral N Shah, Anil Bhansali

**Affiliations:** 1 Departments of Obstetrics and Gynecology Post Graduate Institute of Medical Education and Research (PGIMER) Chandigarh India; 2 Department of Endocrinology Post Graduate Institute of Medical Education and Research (PGIMER) Chandigarh India; 3 Department of Pharmacology Post Graduate Institute of Medical Education and Research (PGIMER) Chandigarh India

**Keywords:** recurrent miscarriage, thyroid peroxidase, hypothyroidism, preterm birth

## Abstract

**Objectives:**

Thyroid antibody positivity during pregnancy has been associated with adverse outcomes including miscarriage and preterm delivery. The aim of the study is to evaluate the obstetric outcome in pregnant women with recurrent miscarriage and their response to levothyroxine (l-T_4_) therapy.

**Study design and methods:**

All pregnant and non-pregnant women between 21 and 35 years of age with a history of two or more consecutive miscarriages were included in the study. A third group comprising 100 pregnant women without a history of miscarriage were taken as healthy controls. Thyroid autoimmunity, prevalence of subclinical hypothyroidism and maternal and foetal complications were analysed in all the groups with appropriate statistical methods.

**Results:**

The mean age of the patients included in the study was 27.0±3.1 years. Of 100 pregnant patients with previous recurrent miscarriage, thyroid autoimmunity (thyroid peroxidase antibody (TPOAb^+^) >34 U/ml) was found in 31% of the cases. The incidence of subclinical hypothyroidism was higher in TPOAb^+^ group than in TPOAb^−^ group (52 vs 16%; *P*=0.0002). There was no difference in the prevalence of miscarriage or obstetric outcomes between recurrent miscarriage and healthy pregnant women group irrespective of TPO status.

**Conclusions:**

The prevalence of thyroid autoimmunity was higher in pregnant women with a history of recurrent abortion compared with healthy pregnant control population. Following l-T_4_ treatment, there was no difference in prevalence of miscarriage between hypothyroid and euthyroid individuals in TPOAb^+^ women.

## Introduction

Miscarriage is the spontaneous loss of the conceptus before 20 weeks of gestation. Potential amount of possible miscarriage before pregnancy is recognised to be about 30%. In clinically recognised pregnancy, it is 10–15% before 8th week and 3% between 8th and 28th weeks. Recurrent miscarriage, defined as loss of two or more consecutive pregnancies (ASRM Practice committee Report, 2008), occurs in ∼1–2% of couples attempting to bear children (1, 2, 3, 4).

Several disorders are known to contribute to recurrent miscarriage including: chromosomal anomalies; anti-cardiolipin antibodies; endocrine disorders such as poorly controlled diabetes mellitus; hyperprolactinaemia and thyroid diseases; and pelvic anatomic abnormalities (5). Recurrent miscarriage can be classified as either primary or secondary. Primary aborters are women who have lost all their pregnancies, whereas secondary aborters have had at least one live born infant.

Autoimmune thyroid disease (AITD) is by far the most frequent cause of hypothyroidism in women of reproductive age. The prevalence of hypothyroidism in the general population of reproductive age is ∼2–3% (6, 7). Overt hypothyroidism is commonly associated with infertility, as thyroid hormones have a direct effect on granulosa cells, luteal cells and oocyte maturation (8). Euthyroid women with thyroid autoimmunity are twice as likely to experience spontaneous miscarriages (9). This probably represents a generalised activation of the immune system, or an increased risk of progression to subclinical hypothyroidism, or it could be due to the transplacental transfer of thyroid receptor blocking antibodies (10, 11). Hence, there is a need to screen for subclinical hypothyroidism and thyroid autoimmunity in pregnancy, especially in women with a history of miscarriages.

However, the management of women with recurrent miscarriage who have thyroid autoimmunity remains controversial. Some investigators recommend the use of empirical thyroxine (T_4_) therapy in women with thyroid peroxidase antibody positive (TPOAb^+^) autoimmunity, although there was no evidence of hypothyroidism (12). A few studies showed that empirical T_4_ therapy in patients with TPOAb^+^ did not improve the obstetrical outcome (13). This study will evaluate the obstetric outcome in pregnant women with recurrent miscarriage and response to levothyroxine (l-T_4_) therapy.

## Research design and methods

One hundred pregnant and 25 non-pregnant women between 21 and 35 years of age with a history of two or more consecutive miscarriages were included in the study. A third group comprising 100 pregnant women without a history of miscarriage were taken as healthy controls. Among the 100 pregnant women with recurrent miscarriages, 85% were recruited in the first trimester and the remaining (15%) in the second trimester. Sixty-seven per cent of patients had primary recurrent abortion while the rest had secondary recurrent abortion. This case–control study was conducted between June 2010 and December 2011 in the Department of Obstetrics and Gynaecology, and the Department of Endocrinology, Postgraduate Institute of Medical Education and Research, Chandigarh, India. It was a prospective study for pregnant women with recurrent abortions and cross sectional for non-pregnant women with a history of recurrent abortions. The study was approved by the Institute's Ethics Committee. All patients provided written informed consent for participation in the trial.

### Eligibility criteria

All pregnant and non-pregnant women with a history of two or more consecutive miscarriages in the age group of 21–35 years were included in the study. Women with known autoimmune disorders, already on treatment for thyroid dysfunction, and a history of cervical incompetence or any other uterine pathology were excluded. All patients underwent a comprehensive medical evaluation including a detailed history and a thorough physical examination.

### Hormonal profile

A 3 ml volume of venous blood was collected in EDTA vacutainers at the initial visit. Each blood sample was analysed for TSH, free tri-iodothyronine (FT_3_), free T_4_ (FT_4_) and anti-TPO by electro-chemiluminescence immunoassay (ELECSYS-2010, Roche–Hitachi Diagnostics). The reference range for the above hormones are as follows: TSH, RR: 0.27–4.2 μlU/ml (inter-assay coefficient of variation (CV) 2.5–3.2%, intra-assay CV 0.5–1.5%); FT_3_, RR: 1.7–4.2 ng/ml (inter-assay CV 1.4–3.2%, intra-assay CV 0.5–2.0%); FT_4_, RR: 0.7–1.8 ng/ml (inter-assay CV 1.0–2.5%, intra-assay CV 1.0–2.5%); and anti-TPO, RR: <34 IU/ml.

All patients with TPOAb^+^ were treated with 25 μg l-T_4_ and titrated according to TSH at the time of recruitment into the study. The patients who had subclinical hypothyroidism were treated as deemed necessary. All patients were followed in the antenatal outpatient department every 4 weeks until 28 weeks. Ultrasonography (USG) was done at 8 weeks in all pregnant women for confirmation of viability and a repeat scan was done around 18 weeks of gestation to find any foetal anomalies. After 28 weeks, they were followed fortnightly until 37 weeks. All patients were monitored for development of any sign or symptom of pre-eclampsia, intrauterine growth retardation (IUGR) or preterm labour. USG for foetal growth and biophysical profile was done as per clinical findings. After 37 weeks, subjects were followed weekly and were delivered according to the obstetric indications. Details of the mode of delivery and any intrapartum or *post partum* complications were noted. After delivery, the birth weight, gestational age and APGAR score at 1 and 5 min, and the presence of any congenital malformations were noted.

Maternal complications were noted as spontaneous abortion, hypertensive complications (gestational hypertension, pre-eclampsia and eclampsia), gestational diabetes mellitus, intrahepatic cholestasis of pregnancy, preterm labour, IUGR, postdatism, preterm premature rupture of membranes and *post partum* haemorrhage. Neonatal outcomes were measured in the form of prematurity (delivery between 20 and 37 weeks), APGAR score, birth weight and congenital malformation.

Twenty-five age-matched non-pregnant women with a history of recurrent abortions were also recruited and investigated for causes of recurrent abortions. Blood samples for thyroid function tests were taken at first visit and treated according to the results. A third group comprising 100 pregnant women without any history of miscarriage were taken as healthy controls. Table 1 compares the baseline characteristics, thyroid hormone profile and maternal–foetal outcome between the pregnant patients of the miscarriage group and the healthy group.

### Statistical analysis

The data have been presented as mean±s.d. if it has a normal distribution; otherwise, median with interquartile range was used. The normality was assessed by Kolmogorov–Smirnov and Anderson–Darling test. Unpaired *t*-test was used for normal data and Mann–Whitney *U* test was used for non-normal data. To analyse the dichotomous data, odds ratio or Fisher's exact test was used. Multivariate analysis was carried out to determine the effect of T_3_, T_4_, TSH and anti-TPO on percentage of live birth per individual. The covariates included for multivariate analysis were age, weight, T_3_, T_4_, TSH, anti-TPO titre, period of gestation and haemoglobin level of the patients. A *P* value of <0.05 was considered significant. All the data were analysed with Minitab 16.00 and SPSS 11.

## Results

The mean age of the women included in the study was 27.0±3.1 years. The odds ratio of having TPOAb^+^ was higher (2.05) in the miscarriage group compared with the healthy group.

Of 100 pregnant patients with previous recurrent miscarriage, thyroid autoimmunity (anti-TPO >34 U/ml) was found in 31% (*P*=0.031) and subclinical hypothyroidism was observed in 27% (*P*=0.74) of the cases. The incidence of subclinical hypothyroidism was higher in the TPOAb^+^ group than in the TPOAb^−^ group (52 vs 16%; *P*=0.0002). TPOAb titre was significantly higher in the hypothyroid group than in the euthyroid with recurrent pregnancy loss group (81.7±132.7 vs 31.9±68.7 IU/ml, *P*=0.016). In the healthy control group, comprised of pregnant women without a history of abortion, the prevalence of subclinical hypothyroidism was 24% and TPO positivity was 18% (*P*=0.70).

There was no difference in the prevalence of miscarriage between hypothyroid and euthyroid individuals in anti-TPO-positive women (*P*=0.23). On linear regression in euthyroid (after exclusion for hypothyroidism) women with anti-TPO positivity, the percentage of live birth was found to correlate with level of TPO antibody (*R*=0.17, *P*=0.01). Similarly, on multivariate linear regression, the percentage of live birth was found to correlate with the level of FT_4_ (*R*=0.17, *P*=0.001). Comparison of TPOAb^+^ and TPOAb^−^ pregnant women with recurrent miscarriage is shown in Table 2. The modes of delivery between the two groups were comparable (*P*=0.7). Among the four aborters in the recurrent miscarriage group, two were in the TPOAb^+^ group and the remaining were in the TPOAb^−^ group with no statistically significant difference (*P*=0.39). Intrahepatic cholestasis was more common in the TPOAb^−^ group (*P*=0.03). The odds ratio of having miscarriage was increased (5.62) when TPOAb^+^ with elevated TSH was compared with normal values.

Twenty-five non-pregnant women with recurrent miscarriage were also analysed for their pregnancy outcome. The mean TSH was 2.7±1.6 μIU/ml and the mean TPO was 28.0±16.8 IU/ml. The prevalence of thyroid autoimmunity and subclinical hypothyroidism in this group was 20% each.

## Discussion

In this study, the prevalence of thyroid autoimmunity was higher in pregnant women with a history of recurrent abortion compared with the healthy pregnant control population. Even though the TSH value was higher in the TPOAb^+^ group than in the TPOAb^−^ group, the prevalence of subclinical hypothyroidism was comparable between the two groups. There was no absolute difference in the prevalence of miscarriage between subclinical hypothyroid and euthyroid pregnant women irrespective of TPO status. However, the odds of having miscarriage in the TPOAb^+^ group increases with rising TSH level compared with normal TSH level. A similar phenomenon was observed with decreasing FT_4_ levels. There was no difference in any of the parameters between pregnant women with recurrent miscarriages compared with non-pregnant women with a history of recurrent abortion.

The prevalence of thyroid autoimmunity in our study was 24% (31% in pregnant women with recurrent abortion) while it was significantly lower (18%) in the healthy group (31 vs 18%, *P*=0.03). The prevalence in the general population described in the literature is 10–15% (14, 15). The odds ratio of having TPOAb^+^ was higher (2.05) in the miscarriage group, indicated a strong association between them. A meta-analysis by Prummel *et al*. (16) showed that TPOAb^+^ was associated with a twofold increased risk of miscarriage as shown in this study. In both the groups, the outcome of current pregnancy was not influenced by TPO positivity or by TSH values. This could be becuase all cases of either isolated TPOAb positivity or of elevated TSH were treated during the course of pregnancy.

Similar results were found by Negro *et al*. (12), who found the miscarriage rate in the TPOAb^+^ group supplemented with T_4_ was comparable to healthy controls (3.5 vs 2.4%). However, unlike our study population, these patients had no history of recurrent miscarriage. None of our patients had isolated hypothyroxinaemia. However, the odds of having the miscarriage were increased with decreasing FT_4_ values. As expected, the mean TSH in the TPOAb^+^ group was higher (3.1±1.9) compared with those in the TPOAb^−^ group (2.0±1.0, *P*<0.001). A possible explanation for high TSH in the TPOAb^+^ group is a reduced functional thyroid reserve associated with chronic autoimmune thyroiditis (17, 18).

In this study, the prevalence of subclinical hypothyroidism was 24% in healthy pregnant women without miscarriage, and 27% in pregnant women with a history of recurrent miscarriage (*P*=0.74). This is higher than the reported prevalence of 10–16% in the literature (14, 15). Such a high prevalence could be due to selection bias, lack of iodine status in our patients, different assay methods and different cutoff levels of TSH used to define subclinical hypothyroid in previous studies.

Women with hypothyroidism have decreased fertility (9), even if they conceive, the risk of miscarriage is increased (19). The risk of gestational hypertension, pre-eclampsia and eclampsia, abruption placentae, *post partum* haemorrhage, premature delivery and IUGR is increased (20, 21). These risks are increased in the whole spectrum of hypothyroidism, including isolated TPO positivity, subclinical hypothyroidism and overt hypothyroidism. In a study by Negro *et al*. (22), there was a positive association between thyroid autoimmunity with preterm delivery and neonatal respiratory distress syndrome in euthyroid women. However, in our study, neither the TPOAb status (positivity or negativity) nor the presence of subclinical hypothyroidism or euthyroidism affected pregnancy outcome. The occurance of intrahepatic cholestasis of pregnancy was higher in the TPO-positive group than in the TPO-negative group (*P*=0.03). There was no difference in prevalence of miscarriage between hypothyroid and euthyroid individuals in TPOAb^+^ women. The prevalence of miscarriage was independent of thyroid status. Similar results were also found with TPOAb^−^ women, when adjusted for age, weight, TFT, TPOAb titre, period of gestation and haemoglobin level.

The miscarriage rate in this study was 4%, which is comparable to a healthy control population. The issue of TPO positivity and the risk of miscarriage in future pregnancies was reported by Pratt *et al*. (10).

Although multiple studies had demonstrated a risk of miscarriage in patients with AITD, the cause has yet to be established (6, 9, 21). TPOAb^+^ is one of the markers of recurrent miscarriage. However, more evidence is needed before dismissing antibody positivity as a cause of adverse pregnancy outcome (22). The association of thyroid autoimmunity and miscarriage could be due to heightened autoimmune imbalance that in turn leads to a greater rejection rate of the foetal graft, and TPOAb^+^ women would tend to become pregnant at an older age (3–4 years older, on average), and older women are more prone to pregnancy loss (23). None of this was found in our study.

Recently, Twig *et al*. (11) described the pathogenesis that underlies infertility and increased pregnancy loss among women with AITD. Thyroid autoantibodies exert their effect in both a TSH-dependent and TSH-independent manner. The latter involves quantitative and qualitative changes in the profile of endometrial T cells, which results in the reduced secretion of IL4 and IL10 together with the hypersecretion of interferon-γ. Polyclonal B-cell activation is two to three times more frequent in thyroid autoimmunity. The hyperactivity and increased migration of cytotoxic natural killer cells which alter the immune and hormonal response of the uterus is up to 40% more common in women with thyroid autoimmunity. Vitamin D deficiency is also linked to infertility and pregnancy loss, suggesting a potential interplay with thyroid autoimmunity in the context of infertility (11).

On multiple liner regression, the percentage of live births was found to correlate with the level of T_4_ (*R*=0.177, *P*=0.012) and anti-TPO (*R*=0.18, *P*=0.015). As expected, the mean TSH in the TPOAb^+^ group was higher (3.1±1.9) compared with that of the TPOAb^−^ group (2.0±1.0). The early evaluation of human thyrocyte TSH receptor stimulating immunoglobulin bioassays with the sera of Graves' disease, Hashimoto's, non-thyroidal auto immune disease and controls, confirms that the cell-based assay of functional immunoglobulin is of clinical importance. It could establish a relationship between the antibodies in question and the disease process (23, 24, 25).

The strength of our study lies in the use of two age-matched control groups, one of non-pregnant women with recurrent miscarriage and another of healthy pregnant women without any history of miscarriage. A single-assay method for TPOAb has been used in the entire study population. The drawbacks of our study were: the small sample size; the fact it is a single-centre study; the lack of a control group that was not treated with l-T_4_; and the unavailability of other antibodies such as anti-thyroglobulin antibody and anti-TSH receptor antibody.

In conclusion, the prevalence of thyroid autoimmunity was higher in pregnant women with a history of recurrent abortion compared with a healthy pregnant control population. Following l-T_4_ treatment, there was no difference in the prevalence of miscarriage between hypothyroid and euthyroid individuals in TPOAb^+^ women. All euthyroid women with thyroid autoimmunity should be treated with l-T_4_ to achieve a favourable maternal and perinatal outcome. A large prospective trial of l-T_4_ therapy in TPO-positive euthyroid women is warranted.

## Figures and Tables

**Table 1:** Comparison of pregnant women with recurrent miscarriage and healthy pregnant control without any miscarriage. Data are expressed as mean±s.d.

**Parameters**	**Pregnant women with recurrent miscarriage** (*n*=100)	**Healthy pregnant control without miscarriage** (*n*=100)	***P* value**
Age (years)	28.0±3.4	27.0±3.1	0.04
Gravida^a^ (range)	4 (3–5)	2 (2–2)	<0.001
FT_3_ a (ng/ml (range))	2.5 (2.1–3.1)	2.7 (2–3.1)	0.76
FT_4_ a (ng/ml (range))	1.2 (1.0–1.4)	1.1 (1.0–1.3)	0.82
TSH^a^ (μIU/ml (range))	2.0 (1.6–2.5)	2.0 (1.4–2.5)	0.58
TPOAb positivity (%)	31	18	0.031
Period of gestation (weeks)	38.4	38.0	0.23
Normal vaginal delivery	75	72	0.63
Operative vaginal delivery	4	4	0.99
Lower segment caesarean section (LSCS)	17	22	0.37
Gestational hypertension	6	4	0.75
Preterm labour	12	16	0.41
IUGR	6	4	0.75
Intrahepatic cholestasis of pregnancy	5	4	0.99
Premature rupture of membrane	1	3	0.62
Birth weight (kg)	2.8	2.8	0.33

a Median.

**Table 2:** Comparison of TPOAb^+^ and TPOAb^−^ pregnant women with recurrent miscarriage. Data are expressed as mean±s.d. *indicates statistical significance (*P*<0.05).

**Parameter**	**TPOAb^+^ group** (*n*=31)	**TPOAb^−^ group** (*n*=69)	***P* value**
Age (years)	26.4±3.0	27.3±3.1	0.1
Free T_3_ (ng/ml)	3.2±2.8	2.5±0.7	0.06
Free T_4_ (ng/ml)	1.2±0.4	1.3±1.0	0.3
TSH (μIU/ml)	3.1±1.9	2.0±0.8	0.001*
TPOAb titre (IU/ml)	105.0±150.6	18.5±9.6	0.001*
Gestation at delivery	38.6±6.7	38.9±4.2	0.7
Mode of delivery			
Normal vaginal delivery	24 (77%)	51 (74%)	0.7
Operative vaginal delivery	1 (3%)	3 (4%)	
Lower segment caesarean section (LSCS)	4 (13%)	13 (19%)	
Missed abortion	2 (7%)	2 (3%)	
Complications			
Abortion	2 (7%)	2 (3%)	0.4
Preterm labour (PTL)	1 (3%)	11 (16%)	0.07
Intrauterine growth retardation	2 (7%)	4 (6%)	0.8
Gestational hypertension	2 (7%)	4 (6%)	0.8
Intrahepatic cholestasis of pregnancy (ICP)	1 (3%)	4 (13%)	0.031*
Postdatism	3 (10%)	3 (4%)	0.2
Preterm premature rupture of membranes	0	1 (1%)	0.5
